# Text-Book of Pathological Anatomy and Pathogenesis

**Published:** 1887-08

**Authors:** 


### Book Reviews.

---

A Text-book of Pathological Anatomy and Pathogenesis. By Ernest Zeigler, Professor of Pathological Anatomy in the University of Tübingen. Translated and edited for English Students by Donald MacAlister, M. A., M. D., Fellow of the Royal College of Physicians, Fellow and Medical Lecturer of St John's College, University Lecturer in Medicine, and Physician to Addenbrooke's Hospital, Cambridge. Three parts complete in one volume. New York: William Wood & Company. 1887. Price, extra muslin, $5.50; sheep, $6.50.

Those who have kept pace with the literature of the day have already seen and examined the parts of this contribution to scientific pathology as they appeared in separate volumes, but it is a most acceptable offering of the publishers to give the whole in one volume, so that the searcher after knowledge may find information upon whatever topic in which he may be interested.

The work is brought up to the latest investigations in this field, and with a thorough but concise digest of the elementary principles in an introduction, it covers all the ground which the student or physician may have occasion to explore.

A work such as this ought to be most acceptable to all classes of the medical profession at present. A transition stage of pathology, from the realm of the obscure and gross changes in structure to the minute and well-defined tissue transformations detected by the microscope, has brought to light the underlying principles involved in disease.

Every practitioner in any department needs to understand the great fundamental truths presented in the introduction of this text-book, and woe be unto the unlucky wight who claims to be a doctor in medicine without being able to give a reason for his faith. We have reached a point in scientific attainments which does not allow any member of the profession to do the heavy standing round, putting on grave, owlish looks and maintaining ominous silence amongst his colleagues. He must be prepared to elucidate the pathological elements complicating a diagnosis, and not depend upon a dogmatical assertion of an opinion without data to support it.

It is by a thorough study of pathological anatomy, as presented in this elaborate treatise, that the physician and surgeon can be prepared to grapple with the intricacies of disease. A cursory perusal of the work will satisfy every one of its enlarged scope of usefulness. In the words of the author, a knowledge of the etiology of the several forms of disease is of the very highest import. If we could but define a disease, not according to its symptoms or its morbid anatomy, but strictly referring it to its causes, we should gain far more than a clear comprehension of the affection; we should have done much to settle the therapeutic treatment. The path along which etiological research has to proceed is twofold—examination of the altered human organ on the one hand, experiment on the other. Experiment is by far the more promising way. We must endeavor to make out experimentally the biological properties of the several fungi as they are found in the human body. But there are sources of disease other than parasitic—such as high temperature, cold, chemically active substances, etc. These must not be overlooked.

Knowledge of the morphology, the genesis, the etiology of morbid changes is the object and aim of pathological anatomy. On comparing the domain of the pathological anatomist with that of the clinical observer, it will not escape our notice that there is a gulf betwixt them. To bridge this gulf is the task of pathological physiology. Through her mediation anatomical research joins hands again with practical medicine. Such are the investigations embodied in this vast collection of erudite observations by Zeigler.

